# Housing conditions and COVID-19 in Barcelona: do they change by gender?

**DOI:** 10.1186/s12889-024-20540-7

**Published:** 2024-11-14

**Authors:** Gloria Perez, Lluís Forcadell-Diez, Alexia Reyes, Catherine Perez, Xavier Bartoll, Carme Borrell

**Affiliations:** 1 Agència de Salut Pública de Barcelona (ASPB), Plaça Lesseps 1, Barcelona, 08023 Spain; 2 CIBER Epidemiología y Salud Pública (CIBERESP), Madrid, Spain; 3 Institut de Recerca Sant Pau, Barcelona, Spain; 4 Universitat Pompeu Fabra, Barcelona, Spain

**Keywords:** Housing, Home environment, COVID-19, Urban health, Health inequities

## Abstract

**Background:**

Evidence has linked poor housing conditions to negative health outcomes. However, in urban contexts characterized by social vulnerability and population-level inequalities, the gender perspective is often overlooked, despite evidence showing that housing conditions impact men and women differently in terms of health. This study aimed to describe the association between housing conditions and the prevalence of COVID-19 among men and women in Barcelona, Spain.

**Methods:**

An observational cross-sectional study was conducted using the 2021 Barcelona Health Survey. The study population consisted of non-institutionalized residents over 14 years of age in Barcelona. The survey was administered to a sample of 3,545 participants during the COVID-19 pandemic, between February 2021 and February 2022. Crude and adjusted prevalence ratios of COVID-19 (aPR), along with 95% confidence intervals (95% CI), were estimated using multivariate Poisson regression models with robust variance. The analysis was stratified by gender.

**Results:**

A significant gradient was observed across age groups for both men and women, with higher COVID-19 prevalence in younger categories. The prevalence was notably higher among individuals born in low-income countries, particularly for women (aPR 1.62). We also identified a significant association between housing conditions, vulnerability factors, and COVID-19 prevalence in both men and women. Living with four or more cohabitants was associated with higher prevalence (aPR 1.96 for women and 1.89 for men), as was the presence of dampness in the home (aPR 1.34 for women and 1.27 for men). Additionally, energy poverty was significantly associated with higher COVID-19 prevalence in women (aPR 1.36), but not in men.

**Conclusion:**

This study highlights the association between housing conditions and COVID-19 in Barcelona, with a pronounced impact on young people, women from low-income countries, and women experiencing energy poverty.

## Introduction

Housing conditions have an impact on physical and mental health and health inequalities [1, 2]. This impact is through four interrelated dimensions: (1) the household which includes aspects related to affordability, stability, security of tenure and emotional attachment to housing (2) the physical housing conditions of the dwelling, (3) the physical environment as green areas and (4) the social environment or community of the neighborhood where the dwelling is located [3].

All these conditions are determined by the housing system of each country, and by other macroeconomic and social policies that affect the quality and access to housing. In addition, the health impacts of housing are influenced by various axes of social inequality, such as gender, age, social class, and racialization, among other factors. Specifically, the review of Vasquez-Vera et al. [4] shows how the health impacts of housing are influenced by gender, with housing-related health outcomes generally being worse for women and the determinants differ for men and women. Regarding age, very few studies analyze vulnerable age groups such as young or the elderly, and the ones that analyze them do not include a gender perspective. The underrepresentation of these age groups may contribute to invisible health inequalities since middle-aged groups are considered as the only subject of research [5]. Also, other factors such as social class [6], country of origin [7] among others, shape people besides gender.

In Spain, the right to housing has been commodified through the neoliberal conception of private property as a tenure regime and consumer good [8]. In addition, Spain has been characterized by a poor commitment to creating and maintaining public housing stock. This has encouraged the accumulation of capital through speculation, construction, and tourism, exacerbating inequalities among social classes [9]. This is most noticeable in large cities and tourist areas. Furthermore, in the last two decades in Barcelona, average house prices, both for sale and rent, have more than doubled in comparison to income. This issue has further exacerbated by COVID-19, making access to housing even more challenging and being a precipitator and outcome of social vulnerability. Another important factor is the increase in rental homes during the pandemic compared with owned homes. There has also been a rise in housing impairment, both due to the need for rehabilitation and the prevalence of substandard housing lacking a certificate of habitability. Residential insecurity has increased, with concerns about rent hikes, non-payment, and evictions [10].

The COVID-19 pandemic has impacted populations worldwide. In the city of Barcelona, the pandemic affected 36% of the city’s population. Throughout the six waves that occurred in the city, revealed significant social inequalities in COVID-19 incidence across different age groups, gender, geographical areas, and income levels [11]. In all countries, but especially in those of low and middle incomes, there is concern regarding the effects of the pandemic on the most deprived populations [12]. These population groups have difficulties in adopting preventive measures (such as social isolation); they are exposed to a context of pragmatic vulnerability that increases the risk of disease infection [13]. The social conditions in which people live and which express, to a greater or lesser extent, the risk of illness, are called social determinants of health (SDH) [14]. In this context, housing conditions are a key element that can either promote or hinder the infection by COVID-19 and the resulting inequalities in infection.

Many factors related to housing conditions such as overcrowding, residential security and neighbourhood characteristics have linked to a higher risk of COVID-19 infection. In this regard, Ahmad et al. [15] established that for every 5% increase in households with poor housing conditions, there was a 50% higher risk of COVID-19 incidence and a 42% higher risk of COVID-19 mortality across US counties. Moreover, the relationship between housing conditions and perceived physical and mental health during lockdown periods is well-documented [14]. Also, pre-existing health conditions that could influence susceptibility to and outcomes of COVID-19 and exacerbated the effects of COVID-19 [16].

Robust literature had linked poor housing conditions to worse health outcomes in the city of Barcelona [17]. Also, it has been established how the availability of appropriate housing facilities has been linked to a decline in infectious diseases including lower respiratory tract infections [18]. The relationship between poor housing conditions and COVID-19 outcomes are currently known in countries with higher incidence and mortality of COVID-19 [15] and also there is evidence pointing to the role of housing conditions in COVID-19 transmission in the municipalities of Brasil [12]. However, in urban contexts characterized by social vulnerability and population-level inequalities, the gender perspective is often not included, despite evidence of the gender-differentiated impact of housing conditions on health. Gender is not systematically considered in the analysis [4]. Therefore, this study aimed to describe the association between housing conditions and the prevalence of COVID-19 for men and women in Barcelona, Spain.

## Methods

This was an observational cross-sectional study. The study population consisted of non-institutionalized inhabitants older than 14 years of the city of Barcelona. The data sourced from the Barcelona Health Survey (BHS) 2021 which is the eighth edition health survey promoted by the Agència de Salut Pública de Barcelona (Public Health Agency of Barcelona, ASPB). It is part of the Statistical Plan of the Generalitat de Catalunya, regional government, and as such, all the requirements of official statistics apply to it, including, among others, the guarantee of confidentiality of the data obtained, which are protected by statistical secrecy.

The statistical universe is 1,653,140 people, the entire non-institutionalized population, residing and registered in the city of Barcelona older than 14 years according to data from the Official Population Register of Barcelona as of January 2021, from which the individuals who habitually live in nurse-houses or other institutions such as collective establishments (e.g., socio-health centers, convents, or military barracks), as well as those who do not live in family households, should be excluded. The selection of the individuals to be interviewed was made through a simple random sampling process, carried out by the Municipal Data Office of the Barcelona City Council, based on the Barcelona Population Register.

The sample unit of the BHS is individuals (not households or families) and the size is 4,000 respondents, distributed across districts of the city. This approach implies that, to analyse results at the city level, the sample has been weighted by applying the corresponding weighting coefficients.

The BHS is a personal face to face and household survey, meaning it is conducted with individuals contacted at their home by professional interviewers. More information about the survey can be obtained by accessing its manual [19].

Data were collected between February 2021 and February 2022 concurrently with the COVID-19 pandemic (March 2020 to March 2022). During the study period, the first wave of the pandemic, which was the most devastating of all three years, had already concluded. Vaccination efforts began in December 2020 and continued throughout the pandemic. Schools reopened in September 2020, and the use of masks became widespread among the city’s population. Throughout the study period, the restrictions imposed by the pandemic gradually became normalized.

### Variables

#### Dependent variable

The dependent variable was “Have you ever been diagnosed with COVID-19?”, measured through a self-reported question with yes/no responses.

The main covariate variables were classified as:

#### Explicative variables

Housing variables:

1. The number of cohabitants asked as: “How many people live in this house on a regular basis?” The numerical answer grouped in none, one, two, three, four, five or more.
2. Housing tenure model asked as: “What the tenancy regime is of your home?”. The possible answers were grouped as: (a) Proprietary (including fully paid, mortgage paid), (b) Rent (including market price or social rent) and (c) Others (including re-renting a part of a flat, rented by social services or NGOs, lent by relatives or friends, another situation).
3. Housing typology derived from two questions: (1) “The apartment is equipped with lift?” with answer yes or no. (2) “Type of dwelling where the interviewee lives”. Answers were grouped as follow: (a) With lift, (apartment building with lift), (b) Without lift (including building with flats without lift, semi-detached single-family house, semi-detached single-family house), c). House (including detached single-family house), and d) Don’t know/don’t answer or another type.
4. Access to outdoor space from the question “Does your home have a balcony or terrace?”. The answer are: (a) Yes, balcony, (b) Yes, terrace for private us, (c) Yes, terrace or communal area, (d) No, neither balcony nor terrace. The answer was grouped as a b, c = yes; d = No.

**2. Vulnerability variables** related to the participant’s housing situation:

1. Energy poverty derived from the question “Can you afford to keep your home at an adequate temperature during the cold months?” with the answer: Yes/no.
2. Overcrowding based on two questions: “How many people live in this house on a regular basis?” and “Approximately how many square meters is your home?” Both answers are numerical. The formula used is: Surface area / Household members ≤ 15 m² per person.
3. Residential insecurity derived from the question “Do you think that in the next 6 months you will be obliged to move house?” Grouped as: Yes (including the following answers: Yes, you will be obliged to change accommodation; and Yes, you plan to change your place of residence) and No (No, you do not expect to change your place of residence).
4. Presence of damp from the question: “Does your home have leaks, dampness in walls, floors, supports or foundations, or rotting of floors, window frames or doors?” With answer: yes/no.

Sociodemographic adjusting covariates were:

1. Age grouped in 15–44, 45–64, 65–74 and 75 years and older.
2. Occupational social class: In the BHS, occupational social class can be obtained with the current or previous occupation of the persona interviewed or if he/she was not working with the occupation of his/her partner or the occupation of the head of the household. Following the proposal of the working group on social determinants of the Spanish Society of Epidemiology, based on the CNO-2011 [20]. Occupational social class was grouped in: Class I: Managers in administration and companies with over 10 employees, requiring advanced degrees. Class II: Managers in companies with under 10 employees, first-degree holders, artists, and athletes. Class III: Administrative, protection, security personnel, self-employed, and manual supervisors. Class IV: Skilled and semi-skilled manual workers. Class V: Unskilled workers.
3. Country of birth grouped as: Catalonia, rest of the Spanish state, high-income countries, and low-income countries according World Bank classification [21].

Health adjusting variables:

1. Number of chronic disorders: The presence or absence of 21 chronic disorders has been grouped into four categories: none, one, two, or three or more [22].
2. Self-perceived health: From 5 categories was grouped as good, fair and poor [23].

Stratification variable: All analysis were stratified using sex/gender, as men and women [24].

### Statistical analysis

This study included a total of 3,545 inhabitants aged 14 and older (1,891 women and 1,654 men). Data analyses involved descriptive univariate and bivariate analyses, as well as multivariate regression analyses. Prevalence was calculated to assess variations in the frequency distribution of COVID-19 across explanatory and adjusting variables. The chi-square test was used to identify significant differences in proportions among groups. The adjusted prevalence ratio of COVID-19 (aPR) and its 95% confidence intervals (95% CI) were estimated using multivariate Poisson regression models with robust variance [25].

According to the study’s conceptual framework, the different models included in the results section show various combinations of covariates. Model 1 includes sociodemographic, housing, and vulnerability variables. In constructing Model 2, health variables were added to Model 1. However, due to the interaction between health variables and age, age groups were excluded from the final Model 2.

The results section presents two multivariable models out of the ten developed for each sex. The models selected were chosen to make the fitted models easier to interpret. Potential multicollinearity was assessed using linear regression and the variance inflation factor. Interactions between variables were also tested. All analyses were stratified by gender, and the analyses were performed using Stata 15.

## Results

The prevalence of COVID-19 was higher in men (16.9%) than in women (14.7%), as shown in Table 1. A significant gradient was observed across age groups, with a higher prevalence of COVID-19 in the younger categories and among individuals born in low-income countries, according to the Chi2 test. Social class was not significantly associated with COVID-19 prevalence.

**Table 1:** Prevalence and adjusted prevalence ratio with 95% confidence interval between housing conditions, social vulnerability and health and the infection by COVID-19 in women and men in Barcelona (Spain). 2021–2022

		WOMEN (N = 1 891)						MEN (N = 1 654)					
		With COVID-19 (*N* = 278)						With COVID-19 (*N* = 279)					
				Model 1		Model 2				Model 1		Model 2	
		n	P*	aPR	95%CI	aPR	95%CI	n	P*	aPR	95%CI	aPR	95%CI
Sociodemographic Variables	**Age**												
	15 to 44 years	147	18.8	1.81	1.03–3.16			173	22.5	2.33	1.32–4.11		
	45 to 64 years	83	14.5	1.65	0.95–2.84			69	13.4	1.47	0.82–2.63		
	65 to 74 years	28	12.3	1.77	0.98–3.19			21	11.7	1.5	0.79–2.86		
	75 years and above	20	6.5	1				16	8.3	1			
	**Social class**												
	I-II	97	14.6	1		1		104	16.4	1		1	
	III	73	14.8	1.11	0.83–1.49	1.07	0.80–1.44	65	18.7	1.1	0.82–1.47	1.09	0.81–1.46
	IV-V	103	15.1	0.89	0.67–1.18	0.83	0.62–1.11	108	16.6	0.86	0.66–1.13	0.86	0.65–1.12
	**Country of birth**												
	Catalonia	136	12.7	1		1		145	15.3	1		1	
	Rest of the Spanish state	38	10.9	1.05	0.72–1.53	0.99	0.69–1.43	35	13.0	1.07	0.74–1.55	0.93	0.65–1.33
	Rest of the high income countries	17	16.4	1.4	0.88–2.21	1.4	0.89–2.22	18	17.1	1.08	0.66–1.75	1.13	0.70–1.83
	Low-income countries	87	23.8	1.62	1.20–2.20	1.65	1.22–2.23	81	24.4	1.37	0.99–1.88	1.36	0.99–1.87
Housing Variables	**Number of cohabitants**												
	None	23	8.8	1		1		16	11.4	1		1	
	Two	76	11.5	1.27	0.78–2.06	1.34	0.83–2.16	82	13.8	1.29	0.75–2.21	1.31	0.77–2.25
	Three	71	16.1	1.59	0.96–2.65	1.73	1.07–2.82	71	17.4	1.41	0.82–2.41	1.61	0.94–2.78
	Four	73	20.1	1.99	1.19–3.31	2.19	1.34–3.57	71	19.8	1.6	0.92–2.77	1.87	1.07–3.25
	Five or more	35	22.4	1.96	1.10–3.48	2.13	1.21–3.74	39	25.8	1.89	1.07–3.35	2.22	1.25–3.95
	**Housing tenure model**												
	Ownership	145	12.8	1		1		144	14.8	1		1	
	Rent	114	18.4	0.92	0.70–1.22	0.95	0.72–1.25	111	19.4	0.9	0.67–1.21	1.01	0.75–1.36
	Other (social. …)	18	15.5	0.9	0.55–1.47	0.92	0.56–1.49	21	22.1	1.24	0.81–1.89	1.36	0.89–2.07
	**Housing typology**												
	Flat with lift	191	13.4	2.95	0.77–11.28	3.04	0.8-11.62	209	16.5	0.81	0.39–1.69	0.88	0.40–1.94
	Flat without lift	66	18.5	3.42	0.89–13.20	3.56	0.92–13.75	59	19.2	0.87	0.40–1.88	0.95	0.42–2.17
	House	2	4.9	1		1		5	20.8	1		1	
	**Outdoor access**												
	Yes	227	14.3	1		1		227	16.3	1		1	
	No	51	16.7	1.06	0.80–1.42	1.07	0.80–1.43	52	20.2	1.15	0.86–1.55	1.19	0.88–1.60
Vulnerability Variables	**Energy poverty**												
	No	216	13.6	1		1		236	16.6	1		1	
	Yes	61	20.9	1.35	1.01–1.79	1.36	1.02–1.82	42	19.4	1.09	0.79–1.50	1.06	0.76–1.46
	**Overcrowding**												
	No	249	14.2	1		1		249	16.6	1		1	
	Yes	29	20.4	0.87	0.59–1.28	0.88	0.60–1.30	30	20.0	0.81	0.54–1.22	0.78	0.52–1.17
	**Residential insecurity**												
	No	237	14.3	1		1		231	16.0	1		1	
	Yes	39	20.0	1.07	0.78–1.45	1.1	0.81–1.50	39	22.4	1.17	0.85–1.62	1.17	0.84–1.62
	**Damp**												
	No	217	13.6	1		1		220	15.8	1		1	
	Yes	61	20.7	1.34	1.01–1.78	1.35	1.02–1.79	59	22.5	1.27	0.96–1.68	1.38	1.04–1.82
**HEALTH** **VARIABLES**	**Number of chronic disorders**												
	None	74	17.6			1		91	16.8			1	
	One	54	16.2			1	0.71–1.40	71	18.9			1.18	0.87–1.58
	Two	44	15.8			0.99	0.69–1.42	44	17.4			1.24	0.87–1.75
	Three or more	106	12.3			0.81	0.58–1.11	73	15.2			1.18	0.85–1.65
	**Self-perceived health**												
	Good	92	15.4			1		131	20.4			1	
	Fair	118	14.1			1.02	0.78–1.34	106	15.0			0.71	0.55–0.92
	Poor	68	14.9			1.2	0.83–1.73	42	13.9			0.74	0.51–1.08

P: Prevalence, defined as the proportion of COVID-19 cases within the category, expressed in %

aPR adjusted prevalence ratio

95%CI Confidence Interval with alpha = 0.05

When analyzing housing variables, the prevalence of COVID-19 was significantly higher among individuals living with five or more cohabitants (women, 22.4%; men, 25.8%) and those residing in rented accommodations (women, 18.4%; men, 19.4%). Regarding vulnerability factors, the variables associated with a significantly higher prevalence of COVID-19 in women included energy poverty (women, 20.9%; men, 19.4%), overcrowding (women, 20.4%; men, 20.0%), residential insecurity (women, 20.0%; men, 22.4%), and the presence of dampness in the home (women, 20.7%; men, 22.5%) (Table 1).

After adjusting model 1 for women, without considering health variables, the prevalence of COVID-19 was significantly higher in women aged 15–44 years compared to those aged 75 years and older (aPR = 1.81, 95% CI: 1.03–3.16) (Table 1).

The prevalence of COVID-19 was significantly higher in women from low-income countries compared to those born in Catalonia (aPR = 1.62, 95% CI: 1.20–2.20). Prevalence was not significantly associated with social class. Among housing variables, the highest prevalence was observed in households with four cohabitants (aPR = 1.99, 95% CI: 1.19–3.31) and in those with five or more cohabitants (aPR = 1.96, 95% CI: 1.10–3.48).

Regarding vulnerability factors, COVID-19 prevalence was significantly associated with energy poverty (aPR = 1.35, 95% CI: 1.01–1.79) and the presence of dampness in the home (aPR = 1.34, 95% CI: 1.01–1.78). In the adjusted Model 2, which included health variables but excluded age groups, the results were similar in both magnitude and direction to those found in adjusted model 1.

In the adjusted model 1 of men without adjusting for health variables, there was a significant association between COVID-19 prevalence and age, with a higher prevalence in the youngest age group (aPR = 2.33 95%CI: 1.32–4.11). No significant associations found in men for social class or country of birth. Among housing variables, only the number of cohabitants was significant, with the highest prevalence being found in residences with five or more cohabitants (aPR = 1.89 95%CI: 1.07–3.35). Among housing variables, the number of cohabitants was significant, with the highest prevalence being found in number of cohabitants of four.

(aPR = 1.87 95%CI: 1.07–3.25) and in those with five or more (aPR = 2.22 95%CI: 1.25–3.95). Among vulnerability variables, the prevalence of COVID-19 was significantly associated with damp (aPR = 1.38 95%CI: 1.04–1.82). It is worthy to highlight that prevalence of COVID-19 is inversely associated with fair self-perceived health (aPR = 0,71 95%CI: 0,55 − 0,92) in men.

## Discussion

The results of this study suggest the presence of associations between housing conditions and the prevalence of COVID-19 in Barcelona, revealing certain gender differences. Variables related to COVID-19 were notably the number of cohabitants and the presence of dampness, affecting both men and women, while energy poverty specifically impacted women. Additionally, younger men and women and migrant women from low-income countries experienced a higher impact, facing housing deprivation such as living in crowded conditions [26], which can contribute to the spread of COVID-19. Furthermore, this population confronts daily life precariousness, closely linked to housing deprivation [27].

The observed association with the number of cohabitants could be attributed to the increased social interactions within the household. Moreover, structural aspects of the dwelling play a role in elevating the prevalence of COVID-19, as seen in issues like dampness, possibly due to their connection to socioeconomic and housing deprivation [28]. Additionally, gender and country of birth function as social determinants, exacerbating health outcomes for women and individuals born in low-income countries, as previously documented [6]. The absence of connection between social class and COVID-19 may be due to the distinct incidence patterns characterising each wave of COVID-19 in Barcelona. Belonging to a lower income-area changed from being a risk factor (waves 1, 2, 4 and 5) to being a protective factor in the sixth wave of the pandemic [29]. Nevertheless, our results show that material conditions significantly contribute to the heightened prevalence of COVID-19, with individuals facing greater vulnerability experiencing a higher incidence of COVID-19 [30].

To analyze how housing conditions impacted men and women differently, we stratified the data by gender. Overall, the determinants were similar for both men and women, with the exception of energy poverty, which was associated with COVID-19 prevalence only in women. This may be related to the fact that there are older women than men, as well as a higher number of women living alone, who likely spend more time at home compared to men. It is important to consider that energy poverty is also linked to overall poverty levels. Additionally, women tend to express greater concern about household dynamics and inadequate living conditions, and they often show more disagreement with government measures [31].

It is noteworthy that during the pandemic, women were more vulnerable to COVID-19 and its consequences due to their overrepresentation in care professions, which increased their exposure to infection. Additionally, women often bear the responsibility for reproductive work, serving as the primary caregivers for those affected by COVID-19 in their households, particularly children and the elderly. This burden was further intensified during lockdowns [17]. A significant finding of this study was that health variables were not clearly associated with a higher prevalence of COVID-19. This may be attributed to the study’s focus, which did not analyze susceptibility to the disease but rather the incidence of diagnoses [17]. We hypothesize that individuals with a higher number of chronic disorders may not have been as exposed to COVID-19, especially if they were aware of their health status. Furthermore, it is essential to consider that the majority of COVID-19 cases were among younger individuals, who typically have fewer chronic conditions.

Overcrowding has been associated with the spread of respiratory illnesses [32]. Also, the WHO published the guidelines about housing and health identifying how poor housing is related with environmental risk factors including overcrowding, air and water quality and lack of. access to adequate plumbing and sanitation, as factors contributing to the burden of infectious diseases including airborne respiratory illnesses [18].

The study’s limitations primarily arise from the utilization of housing variables available in the BHS. While having more detailed information on housing conditions would have been advantageous, the survey’s inherent nature limited the inclusion of additional data. Moreover, surveys that simultaneously incorporate both health and housing variables are infrequent. Therefore, the BHS provided a unique opportunity to delve into a topic that is underrepresented in scientific literature.

The BHS does not include the non-institutionalized population, meaning it does not account for those who suffered the most during the first wave of the pandemic. Regarding the risk of COVID-19 among institutionalized individuals, it is important to note that during the survey period (February 2021 to February 2022), the first wave of the pandemic— which was the most devastating and severely impacted older adults in nursing homes— had already concluded. Additionally, it is noteworthy that just before the survey was conducted (in December 2020), vaccination efforts began for institutionalized older adults as a prioritized group. Vaccination continued for the rest of the population throughout 2021 and beyond.

Moreover, it is essential to highlight that the question from which the dependent variable was derived asked whether respondents had ever been diagnosed with COVID-19 up to the time the survey was conducted. Consequently, the institutionalized population was not included in this analysis.

As we described in the introduction, social inequalities were evident throughout the pandemic in the city; however, the data indicate that incidence rates reversed during the last two waves, with higher-income areas experiencing the highest incidence. Additionally, socioeconomic inequalities in COVID-19 incidence varied by wave, age group, and phase of the pandemic in the city of Barcelona [29].

In conclusion, this study highlights the association between housing conditions and COVID-19 in Barcelona, with a pronounced impact on vulnerable populations, particularly young people, women from low-income countries, and women experiencing energy poverty. These findings underscore the crucial importance of considering these factors in the development of housing policies and in formulating strategies to effectively address and mitigate the impact of future pandemics.

## Data Availability

Data of this research can be obtained asking by mail to the Barcelona Agency of Public Health at https://www.aspb.cat/contacte/.

## Abbreviations

ASPB: Agència de Salut Pública de Barcelona (Public Health Agency of Barcelona)
aPR: Adjusted prevalence ratio
BHS: Barcelona Health Survey
CNO: Clasificación Nacional de Ocupaciones (Nacional classification of occupation)
CI: Confidence interval
P: Prevalence

## References

[1] Bolte G, Braubach M, Chaudhuri N, Deguen S, Fairburn J, Fast I et al. Environmental Health Inequalities in Europe. WHO report. 2012. 212 p.

[2] Vásquez-Vera H, León-Gómez BB, Palència L, Pérez K, Borrell C. Health Effects of Housing Insecurity and Unaffordability in the General Population in Barcelona, Spain. J Urban Heal. 2021;98(4):496–504. 10.1007/s11524-021-00546-x PMC8382804 34231119

[3] Novoa AM, Bosch J, Díaz F, Malmusi D, Darnell M, Trilla C. El impacto de la crisis en la relación entre vivienda y salud. Políticas de buenas prácticas para reducir las desigualdades en salud asociadas con las condiciones de vivienda. Vol. 28, Gaceta Sanitaria. 2014. pp. 44–50. 10.1016/j.gaceta.2014.02.018 24863993

[4] Vásquez-Vera C, Fernández A, Borrell C. Gender-based inequalities in the effects of housing on health: A critical review. Vol. 17, SSM - Population Health. 2022. p. 101068. 10.1016/j.ssmph.2022.101068 PMC8961216 35360438

[5] Ayalon L, Tesch-Römer C. Introduction to the section: Ageism—Concept and origins. Contemporary perspectives on ageism. 2018. 1–10 p.

[6] Wilkinson E, Ortega-Alcázar I. Stranger Danger? The Intersectional Impacts of Shared Housing on Young People’s Health &Wellbeing. Heal Place [Internet]. 2019;60:102191. https://www.sciencedirect.com/science/article/pii/S135382921830902X 10.1016/j.healthplace.2019.102191 31678830

[7] Krieger N, Smith K, Naishadham D, Hartman C, Barbeau EM. Experiences of discrimination: validity and reliability of a self-report measure for population health research on racism and health. Soc Sci Med. 2005;61(7):1576–96. 16005789 10.1016/j.socscimed.2005.03.006

[8] Rodríguez AR. La política De vivienda en España en El contexto Europeo. Deudas y retos. Rev INVI. 2010;25(69):125–59.

[9] Andrews D, Caldera Sánchez A, Johansson A, OECD Economics Department Working Papers. Housing Markets and Structural Policies in OECD Countries [Internet]. Vol. 836,. 2011. https://www.oecd-ilibrary.org/economics/housing-markets-and-structural-policies-in-oecd-countries_5kgk8t2k9vf3-en

[10] Observatori Metropolità de l’Habitatge de Barcelona. L’habitatge a la metròpoli de Barcelona en el 2021. Entre la COVID-19 i la crisi inflacionista [Internet]. Barcelona. 2022. https://www.ohb.cat/wp-content/uploads/2022/11/O22008_SI_Informeanual_2021.pdf

[11] Agencia de Salut Publica de Barcelona. #COVID19aldiaBCN [Internet]. 2020. https://aspb.shinyapps.io/COVID19_BCN/

[12] De Souza CDF, Machado MF, Do Carmo RF. Human development, social vulnerability and COVID-19 in Brazil: a study of the social determinants of health. Infect Dis Poverty. 2020;9(1). 10.1186/s40249-020-00743-x PMC7456757 32867851

[13] Ahmed F, Ahmed N, Pissarides C, Stiglitz J. Why inequality could spread COVID-19. Lancet Public Health. 2020;5:e240. 32247329 10.1016/S2468-2667(20)30085-2 PMC7270465

[14] Vásquez-Vera H, León-Gómez BB, Borrell C, Jacques-Aviñó C, López MJ, Medina-Perucha L, et al. Inequities in the distribution of COVID-19: an adaptation of WHO’s conceptual framework. Gac Sanit. 2022;36(5):488–92. 34823902 10.1016/j.gaceta.2021.10.004 PMC8526437

[15] Ahmad K, Erqou S, Shah N, Nazir U, Morrison AR, Choudhary G, et al. Association of poor housing conditions with COVID-19 incidence and mortality across US counties. PLoS ONE. 2020;15(11 November):e0241327. 33137155 10.1371/journal.pone.0241327 PMC7605696

[16] Horton R, Offline. COVID-19 is not a pandemic. Lancet. 2020;396(10255):874. 32979964 10.1016/S0140-6736(20)32000-6 PMC7515561

[17] Marí-Dell’olmo M, Gotsens M, Pasarín MI, Rodríguez-Sanz M, Artazcoz L, de Olalla PG, et al. Socioeconomic inequalities in COVID-19 in a European urban area: two waves, two patterns. Int J Environ Res Public Health. 2021;18(3):1256. 33573323 10.3390/ijerph18031256 PMC7908269

[18] World Health Organization. WHO Housing and health guidelines [Internet]. 2018. 149 p. https://apps.who.int/bookorders18157_WHOHousingandHealthGuidelines_160x240mm

[19] Bartoll-Roca X, Perez K, Artazcoz L. Manual metodològic de l’Enquesta de Salut de Barcelona 2021 [Internet]. Barcelona, Spain; 2021. https://www.aspb.cat/wp-content/uploads/2022/11/ASPB-Manual-Enquesta-Salut-2021.pdf

[20] Domingo-Salvany A, Bacigalupe A, Carrasco JM, Espelt A, Ferrando J, Borrell C. Propuestas De clase social neoweberiana y neomarxista a partir de la Clasificación Nacional De Ocupaciones 2011. Gac Sanit. 2013;27(3):263–72. 23394892 10.1016/j.gaceta.2012.12.009

[21] World Bank. New country classifications by income level: 2016–2017. 2016.

[22] Beckett M, Weinstein M, Goldman N, Yu-Hsuan L. Do health interview surveys yield reliable data on chronic illness among older respondents? Am J Epidemiol. 2000;151(3):315–23. 10670557 10.1093/oxfordjournals.aje.a010208

[23] Pietz K, Petersen LA. Comparing self-reported health status and diagnosis-based risk adjustment to predict 1- and 2 to 5-year mortality. Health Serv Res. 2007;42(2):629–43. 17362210 10.1111/j.1475-6773.2006.00622.x PMC1955361

[24] Rioux C, Paré A, London-Nadeau K, Juster RP, Weedon S, Levasseur-Puhach S, et al. Sex and gender terminology: a glossary for gender-inclusive epidemiology. J Epidemiol Community Health. 2022;76(8):764–8. 10.1136/jech-2022-219171 35725304

[25] Espelt A, Marí-Dell M, Penelo E, Bosque-Prous M. Resumen Abstract Applied prevalence ratio estimation with different regression models: an example from a cross-national study on substance use research Estimación de la Razón de prevalencia con distintos modelos de Regresión: Ejemplo De Un Estudio Interna. Adicciones. 2016;29(–):105–12. 27391846 10.20882/adicciones.823

[26] Kjøllesdal M, Skyrud K, Gele A, Arnesen T, Kløvstad H, Diaz E, et al. The correlation between socioeconomic factors and COVID-19 among immigrants in Norway: a register-based study. Scand J Public Health. 2022;50(1):50–60. 10.1177/14034948211015860 PMC8807998 33983088

[27] Hick R, Pomati M, Stephens M. Severe housing deprivation in the European Union: a joint analysis of measurement and theory. Soc Indic Res. 2022;164(3):1271–95. 36039339 10.1007/s11205-022-02987-6 PMC9401192

[28] Novoa AM, Ward J, Malmusi D, Díaz F, Darnell M, Trilla C et al. How substandard dwellings and housing affordability problems are associated with poor health in a vulnerable population during the economic recession of the late 2000s. Int J Equity Health [Internet]. 2015;14(1):1–11. 10.1186/s12939-015-0238-z 10.1186/s12939-015-0238-z PMC4632653 26530721

[29] Martinez-Beneito MA, Marí-Dell’Olmo M, Sánchez-Valdivia N, Rodríguez-Sanz M, Pérez G, Pasarín M, Rius C, Artazcoz L, Prieto R, et al. Socioeconomic inequalities in COVID-19 incidence during the first six waves in Barcelona. Int J Epidemiol. 2023;52(6):1687–95. 37494962 10.1093/ije/dyad105

[30] Fernández-Barrés S, Perez G, Piñero M, Reyes A, Pérez K, Artazcoz L et al. Effect of COVID-19 prevention as part of an urban renewal programme. Public Health [Internet]. 2023 Oct 1 [cited 2023 Sep 4];223:179–82. https://linkinghub.elsevier.com/retrieve/pii/S0033350623002548 10.1016/j.puhe.2023.07.014 37666182

[31] López-Contreras N, López-Jiménez T, Horna-Campos OJ, Mazzei M, Anigstein MS, Jacques-Aviñó C. Impact of COVID-19 lockdown on self-perceived health in Chile by gender. Gac Sanit. 2022;36(6):526–33. 35589458 10.1016/j.gaceta.2022.04.002 PMC9050586

[32] Shereen MA, Khan S, Kazmi A, Bashir N, Siddique R. COVID-19 infection: Origin, transmission, and characteristics of human coronaviruses. J Adv Res [Internet]. 2020;24:91–8. 10.1016/j.jare.2020.03.005 10.1016/j.jare.2020.03.005 PMC7113610 32257431

